# *In Vitro* Selection of Cancer Cell-Specific Molecular Recognition Elements from Amino Acid Libraries

**DOI:** 10.1155/2015/186586

**Published:** 2015-09-07

**Authors:** Ryan M. Williams, Letha J. Sooter

**Affiliations:** ^1^Memorial Sloan Kettering Cancer Center, Molecular Pharmacology & Chemistry, 1275 York Avenue, New York, NY 10065, USA; ^2^Basic Pharmaceutical Sciences, West Virginia University, 1 Medical Center Drive, P.O. Box 9530, Morgantown, WV 26506, USA

## Abstract

Differential cell systematic evolution of ligands by exponential enrichment (SELEX) is an *in vitro* selection method for obtaining molecular recognition elements (MREs) that specifically bind to individual cell types with high affinity. MREs are selected from initial large libraries of different nucleic or amino acids. This review outlines the construction of peptide and antibody fragment libraries as well as their different host types. Common methods of selection are also reviewed. Additionally, examples of cancer cell MREs are discussed, as well as their potential applications.

## 1. Introduction

**Figure 1:**
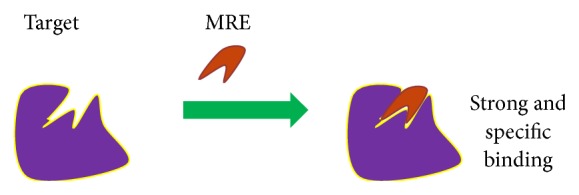
Cartoon explanation of molecular recognition element (MRE) binding. A MRE is any molecule with strong and specific binding to a target of interest.

The systematic evolution of ligands by exponential enrichment (SELEX) is a method of obtaining molecular recognition elements (MREs) (Figure 1) that bind to a target of interest. This* in vitro* process is a powerful tool for selecting molecules useful in the specific detection or treatment of diseases. Selectivity for a certain molecule or disease state can be ensured by designing the experiment with the expected use of the final product being considered, such as buffering conditions, temperature, or environmental complexity.

The SELEX method obtains one or a few molecules that bind to the target with high affinity and specificity. These MREs are selected from a large library of 10^9^–10^15^ random molecules through iterative library screening. SELEX was originally described independently by the laboratories of Gold and Szostak to select nucleic acid MREs that bind to proteins and organic dyes [1, 2]. Since then, MREs have been selected that recognize a range of targets, from single ions and small molecules to nanoparticles and proteins [3–6]. The same SELEX principle has been used to develop antibody fragment and peptide MREs that bind to small molecules, proteins, nucleic acids, and nanoparticles [7–10]. The wide variety of MRE libraries and potential targets speaks to the numerous applications of the SELEX process.

MREs have also been selected that bind to molecules displayed on the surface of cells. Originally performed by the Gold laboratory, SELEX has been used to identify nucleic acid MREs that bind to the surface of red blood cells [11]. This work showed the ability to select MREs that bind to molecules displayed on cell surfaces in their natural state. Since that time, SELEX has been used to select MREs that bind to molecules that are differentially expressed on the surface of cells. MREs have also been selected that bind specifically to the vasculature of rat brain glioblastoma without binding normal rat brain vasculature [12]. Other nucleic acid MREs selectively recognize and bind to molecules that are expressed on the surface of cancer cells [13–15]. This type of MRE selection provides the dual benefits of binding to cell surface molecules and taking advantage of the differences in surface expression between cells.

**Figure 2:**
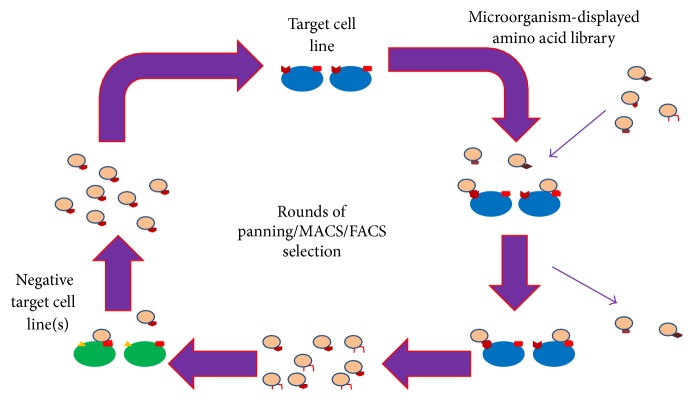
Differential cell SELEX overview. A target cell type is incubated with a naïve amino acid library displayed on the surface of a host. Those that do not bind are removed by various screening methods, commonly panning, MACS, or FACS. Those that do bind are amplified and incubated with negative target cells. Those that do not bind are amplified and resubjected to the target cell line. This iterative screening process continues for multiple rounds under increasingly stringent conditions until one or a few strongly binding MREs are obtained. Figure adapted from [75].

The process of differential cell SELEX makes use of a large, random library of molecules that are incubated with the cell target of interest (Figure 2). Those molecules that bind to the target are retained and amplified, while those that do not are eliminated. The amplified binding molecules are then incubated with the negative target cell lines. Those molecules that do bind these cells are discarded and those that do not are amplified and resubjected to the target cells. This process of targeting and negative targeting is performed (rounds of selection) until an enriched pool of high-affinity binding molecules is achieved. The iterative nature of the SELEX process differentiates it from typical amino acid library screens where only one or a few coincubations occur. Amplification occurs via the polymerase chain reaction (PCR) for nucleic acids and by host replication for amino acid libraries (see Section 2.1) [16–20]. Nucleic acid MREs, however, are less stable and may be degraded if introduced* in vivo* for detection or therapeutic purposes.

Compared to nucleic acids, amino acid-based MREs potentially provide stability and specificity* in vivo* (Table 1). Additionally, the nonreversible nature of their target interaction and high binding affinity is an advantage in tumor cell targeting. While having the positive attributes of antibodies* in vivo*, these MREs are selected* in vitro*. Therefore, more control over their binding target is possible for the experimenter. This includes the ability to preferentially select molecules which bind to a target but not closely related variants. Additionally,* in vitro* selection allows facile identification of binding elements for poorly immunogenic targets which may not be possible with traditional antibody development [21]. Differential cell SELEX takes advantage of differential surface molecule expression for MRE binding. The result of amino acid MRE selection using differential cell SELEX is a molecule that has the advantages of antibodies with tunability in the experimental design.

This review focuses on the methodologies involved in constructing an amino acid library for SELEX, as well as techniques allowing the separation of binding and nonbinding molecules in cell SELEX. Additionally, it gives examples of cell SELEX experiments performed using these libraries. Finally, a discussion of applications for selected amino acid MREs in disease detection and therapeutics is presented.

## 2. Methodology Overview

### 2.1. Library Construction

Amino acid libraries used in SELEX are typically displayed on the surface of living host cells, though there are exceptions. Generally, genetic information that encodes surface expression of the randomized library is inserted into phage, bacteria, yeast, or mammalian cells, though other options exist [22]. Amplification is performed by the natural replication of the host. Between every few rounds of selection, the diversity of the enriched library is assayed by DNA sequencing of the amino acid library-encoding plasmid.

Library construction varies for the type of library. The DNA encoding for peptide libraries typically comes from chemically synthesized randomized DNA libraries [23] or from codon phosphoramidites [24]. Antibody fragment library DNA typically comes from PCR-amplified immune or naïve human antibody-coding cDNA [25]. These are often linked by scaffold sequences for structure and stability. Additionally, they often include DNA restriction enzyme recognition sequences for cloning [26]. Further randomization can be performed by error-prone PCR and shuffling of the encoding fragments [27, 28]. These strategies are often used in affinity maturation of* in vitro* selected binding molecules. Thus, library construction techniques can be utilized for naïve libraries as well as those generated from immunized B cells.

### 2.2. Phage Display

Bacteriophage are the most widely used host for displaying amino acid libraries (Table 2) [29]. Libraries of short, random peptides that are displayed on the surface of phage have been used for SELEX experiments and are commercially available (e.g., Phage Display Peptide Library from New England Biolabs; Beverly, MA). These typically consist of 7–15 random amino acids displayed by fusion to a surface protein. Examples of this include the pIII protein of M13 phage which is necessary for phage infection of* Escherichia coli* [30] and a peptide library displayed on T7 lytic phage, available commercially from EMD Millipore (Darmstadt, Germany) [23]. Random nucleic acid codons are synthesized and inserted into a plasmid for protein fusion. These libraries often consist of approximately 10^9^ different random peptides, all 7–15 amino acids in length.

Additionally, single chain fragment variable (scFv) antibody fragment library, which is a fusion of heavy and light chain antibody antigen-binding regions, has been constructed that is displayed on M13 phage [31, 32]. An antibody fragment (Fab) library, which consists of an antibody's antigen-binding domain in the form of the light chain and half of the heavy chain, and a human heavy chain variable fragment (VH) library, which is only the antigen-binding domain from the heavy chain, have also been constructed that are fused to the surface of bacteriophage lambda [33, 34]. In this case, genetic information from nonimmune human antibody fragments is amplified from donors and constructed into a vector which encodes the fusion protein on the virus surface. It has been noted that phage lambda is more capable of displaying large antibody fragments than M13 [35]. Furthermore, a vaccinia virus infecting yeast has been used to display an antibody fragment library similar to phage display [36].

Phage-displayed amino acid libraries offer the advantages of rapid screening and amplification for the SELEX process coupled with careful outlines of their use [37]. Selected phage is incubated with* E. coli *and plaques are produced. The replicated phage is then subjected to the next round of selection. Technical challenges of phage display systems include inefficient or incomplete display of surface proteins due to reading frame errors, difficulty in expressing large proteins, and the inability to perform posttranslational modifications necessary for eukaryotic proteins [29]. The method used for SELEX is often panning, whereby the library is incubated with immobilized target cells directly in the tissue culture dish (see Section 2.8). While rapid, this method is not as efficient at isolating specific molecules as fluorescent activated cell sorting (FACS), which also provides more quantitative monitoring of enrichment (see Section 2.8). Rapid and efficient amplification coupled with detailed protocols allow phage-displayed amino acid libraries to be useful tools in cell SELEX.

### 2.3. Bacterial Display

Another platform for amino acid library display is through the use of bacterial hosts (Table 2). A peptide display library of 12 random amino acids has been displayed as fusions to the* E. coli* flagellin protein [38]. This library contains approximately 5 × 10^10^ different peptides and is available commercially (Invitrogen; Grand Island, NY). A library of the same size consisting of 15 random amino acids has been displayed as a fusion to the* E. coli* outer membrane protein A (OmpA) [39]. These libraries offer the rapid and efficient screening necessary to select a specific MRE.

Antibody fragment display has also been performed in bacterial systems. A sublibrary of scFv molecules in* E. coli* was generated by PCR randomization of immune antibody fragment-coding DNA sequences [40]. Additionally, scFv molecules have been expressed as fusions to the surface protein A of Gram-positive* Staphylococcus carnosus* [41]. The first screening of a large library of antibody fragments on a bacterial surface has been performed recently [42]. More widespread use of similar libraries is likely to occur in the near future.

Bacterial display of amino acid libraries provides the benefit of rapid screening and amplification as detailed in published protocols [43]. Furthermore, they can be used in both panning and FACS-based separation [44]. Limitations of bacterial display systems include a lack of modularity in construction of various library types on different hosts and scaffolds. These are in addition to potential membrane fusion, difficulty in large protein expression, and an inability to perform posttranslational modifications [45]. While not as widely used as phage libraries, these libraries do not require multiple hosts, therefore increasing speed of the selection. These benefits render bacterial display of amino acid libraries a potentially useful avenue for developing new MREs.

### 2.4. Yeast Display

Yeast display of amino acid libraries is a widely used platform for generating MREs (Table 2). A library consisting of approximately 10^7^ random twelve-amino-acid peptides has been constructed and has proven to be of use in the selection of MREs [46]. It was formed as a C-terminal fusion to the Aga2p protein that is expressed on the surface of* Saccharomyces cerevisiae*. Additionally, there are protocols available to transfer a phage-displayed library to a yeast-displayed format in order to benefit from the advantages of both systems [47].

The use of* S. cerevisiae* to display antibody fragments has also been extensively explored. This microorganism has been used to display a scFv library of approximately 10^9^ different antibody fragments [26]. This library is freely available from the Pacific Northwest National Laboratory (PNNL; Richland, WA). A Fab sublibrary has also been constructed that is displayed on the surface of* Saccharomyces* [48] in addition to antibody fragments displayed on the surface of* Pichia pastoris* [49].

Yeast display of amino acid libraries offers the advantages of use in FACS, as most include sequences for fluorescent protein tagging. Importantly, eukaryotic yeasts have the ability to perform posttranslational modifications not available in bacteria or phage. This is important for expressing the full diversity of antibody fragment libraries [50]. The described yeast systems also offer easy secretion and purification of selected MREs. Some yeast systems, however, have been found to display high levels of poorly folded proteins instead of highly stable structures [51]. Additionally, poor yeast transformation efficiency often limits library size [52]. Nevertheless, the positive attributes allow the use of yeast for selecting MREs by cell SELEX, and protocols for constructing and using these libraries are readily available [53–55].

### 2.5. Mammalian Cell Display

Display of amino acid libraries on the surface of mammalian cells has also been used recently. A peptide library has been constructed that is displayed as a fusion to the CCR5 receptor of human T cells [56]. This library was used to select a binding ligand and also serves a proof-of-principle for the concept.

Antibody fragment libraries have also been constructed for display on mammalian cells. Human HEK-293T, T cells, and B cells have all been used to display scFv libraries [57–59]. Additionally, a novel variation of this type of library has used a eukaryotic retrovirus to infect mammalian cells with a scFv library for replication and display [60]. While these libraries are not commercially available, methods for constructing them are [61].

Mammalian cell display offers the advantage of posttranslational modifications beyond those available in yeast [61]. Thus, the selected amino acid MRE is likely to be functional* in vivo* or for other applications. Technical limitations of mammalian cell display systems include their relatively slow growth rate and their ability to undergo apoptosis [22, 62]. Additionally, mammalian cells exhibit variability in expression levels and are difficult to stably transfect [22]. While these libraries have been constructed, they are not as widely studied as other cellular library hosts.

### 2.6. Ribosome/mRNA Display

One of the more common alternate library hosts is ribosome or mRNA display [63, 64]. Ribosome display links the amino acid library directly to the ribosome and the encoding mRNA. Alternatively, in mRNA display, translated mRNA is covalently linked to the protein through an adaptor molecule [65]. These techniques have each been used to display and select peptides and antibody fragments [64, 66, 67]. Each of these techniques offers the advantages of removing the need for cellular transformation and they allow for easy mutagenesis for PCR. A major disadvantage of both techniques is that selection conditions are limited by those that keep the display complex intact, as proteins and RNA must both be stabilized with degradation minimalized [68]. Additionally, they are limited by protein size (typically less than 300 amino acids), display efficiency, and an inability to display membrane-bound proteins [68].

### 2.7. Target Cells

The state of the target cells in the SELEX process will alter the final product [69]. The use of fixed versus live cells may have consequences in the final success and application of the selected MREs. Fixation by paraformaldehyde (PFA) causes protein cross-linking, though it makes cells easier to work with as they will not degrade during the SELEX incubation period. This clearly would have an effect on experiments designed to obtain cell surface protein-binding molecules. There are examples, however, of MREs selected on live cells that also bind to fixed cells, though this cannot be assumed [70, 71]. There are also examples of MREs selected on live or fixed cells sharing binding motifs but not full sequences [72]. An additional factor may be the use of adherent cell dissociation methods. The use of enzymatic cellular detachment solutions (e.g., trypsin) digests cell surface proteins, which would limit the success of cell SELEX. To circumvent this, investigators often use ethylenediaminetetraacetic acid (EDTA) [73], brief trypsinization [74], or other proprietary nonenzymatic dissociation reagents (e.g., CellStripper; MediaTech, Manassas, VA) [75]. Ultimately, it is most important to consider the desired final application: if the experimenter desires a MRE that binds to fixed tissue sections, fixation would be acceptable; however if binding to live cells* in vivo* or* in vitro* is desired, utilizing live cells is preferable.

### 2.8. Separation Methods

A necessity of the SELEX process is separating molecules that bind to the target from those that do not. In cell SELEX, this is typically done by panning, magnetic sorting, or FACS library screening methods. Often, a combination of these methods is used in order to most efficiently select MREs with high-affinity binding (Table 3) (e.g., [75]). Typically, initial rounds of selection are used to remove molecules with poor solubility, affinity, or cross-reactivity [53]. Initial use of stringent separation methods such as FACS would be unsuccessful due to poor efficiency at low concentrations of the binding population [76, 77]. Thus, initial removal of nonbinding molecules increases the concentration of binding molecules in the library, allowing successful isolation by more stringent methods such as FACS due to its single-cell and quantitative nature, as has previously been described [77, 78]. Additionally, the initial library size is typically much greater than what can be separated by FACS in a reasonable time span. Thus, selections are often individually optimized and often include multiple separation methods.

Panning, as referred to in this review, is a specific screening method of incubating the library with the immobilized target cells directly in the tissue culture well or flask [79]. The unbound library molecules are simply aspirated or decanted. The flask is washed typically three times taking care not to detach the target cells. The bound molecules are then amplified by replication of their host. This process is most thoroughly described for phage-displayed libraries [80, 81] but has also been explored for yeast libraries [53, 75]. For phage, the bound molecules must be eluted and plated on bacteria for replication. In bacterial or yeast libraries, however, the appropriate microbiological media can be added to the cell culture flask or the human cells can be scraped from the flask. The enriched library-containing bacteria or yeast will amplify in this media, while any remaining human cells will not survive under these conditions. For negative selection steps, the unbound library molecules are removed by careful pipetting. The collected host organisms are then placed under optimal growth conditions. This method of separation is robust for large libraries. However, flocculation of the host organisms or inefficient removal of nonbinding molecules is possible. This creates an enriched library that contains some molecules that do not bind to the target. Therefore, panning is an efficient method of initial screening for the first few rounds of SELEX.

Magnetic sorting is another approach of separating binding from nonbinding molecules. Often, magnetic activated cell sorting (MACS) is used as a preenrichment procedure. In the case of noncellular targets, such as proteins, the library is typically passed through a column of magnetic beads on which the target is immobilized [82]. A variation of this method used a B-cell antigen to bind chronic lymphocytic leukemia cells to magnetic beads for use with a phage library [83]. The library molecules bound to the target are then eluted and replicated under appropriate conditions (or unbound molecules for negative selections) (see [84] for in-depth schematic). While useful for robust screening, this method may be difficult for use in cell SELEX due to limited space within the column when considering human cells and library host cells are present. Therefore this method requires more preparation and greater potential for failure, while performing the same preenrichment task as panning. It is for these reasons that the use of MACS for cell SELEX in obtaining amino acid MREs is rare in the literature.

Fluorescent activated cell sorting (FACS) is often used to select MREs that bind to molecules on the surface of cancer cells (e.g., [75]). This method uses dual fluorescent labeling of the library and the target cells, which are removed from the culture flask. Following co-incubation, this mixture is subjected to FACS, wherein the software is instructed to collect events corresponding to double fluorescence. For negative targeting, collecting single fluorescent events corresponding to the library alone will render molecules that do not bind to the target. A yeast-displayed scFv library and a bacterial-displayed peptide library have both been used for cell SELEX through the FACS method [85–87]. The greatest advantage of FACS is the tunability of separation parameters and extremely efficient enrichment of binding molecules. It does, however, take considerably longer to screen very large numbers of cells. This is exacerbated when using two largely different cell sizes, as is the case with human and bacterial or yeast cells. Also, expensive equipment and significant expertise in operation are necessary. Therefore, FACS is more efficiently used after the library has undergone initial enrichment and it is not necessary to use a large number of host cells to ensure full diversity is represented.

## 3. Cell SELEX Results

### 3.1. Peptide MREs

Molecular recognition elements selected from peptide libraries have been extensively explored. Particularly from phage display libraries, the cell SELEX procedure has been used to select MREs that bind specifically to tumor cells. Peptides are short sequences generally less than 25 amino acids. Libraries with a very large diversity can be formed drawing from the inherent diversity of the 20 amino acids. A theoretical maximum for a seven-amino-acid library is 1.28 × 10^9^ or ~10^32^ (20^25^) for a 25-amino-acid library, although typical construction techniques and reasonable working volumes typically limit library size to around 10^10^ molecules. Additionally, there is some bias in insertion of particular amino acids [88]. A breakthrough in peptide library construction was the introduction of codon phosphoramidites (Glen Research; Sterling, VA), which ensures no premature stop codon or insertional biases are present [24]. Compared to antibody fragment libraries, there may be less inherent structure within the MRE, but randomness is ensured.

Peptide MREs in cell SELEX have largely been selected through phage display methods. An example of this is the selection of peptide MREs that bound to the B-cell lymphoma line A20 [89]. This experiment did not use differential cell SELEX, but those MREs were able to differentiate lymphoma cells from other normal white blood cells. In another study, primary chronic lymphocytic leukemia cells were the targets of phage selection using a modified MACS protocol [83]. This study also did not use negative cell lines; however a panel of peptide MREs was selected, some of which were specific to the target cells. A study performed that selected peptide MREs for non-small cell lung cancer cell lines also did not use a negative target cell [90]. One of the selected peptides, however, was successfully used for* in vivo* chemotherapeutic delivery studies. It is therefore possible to select useful peptides without a differential component of the selection.

The likelihood of selecting one or a few specific MREs that are fit for their expected use is increased if negative targets are used. One study selected for peptides that bound to the breast cancer cell line BT-474 but not the benign cell line MCF-10A [91]. This study even did a negative selection against the tissue culture flask itself. This, however, seems to be inherent if negative selections are performed in the same type of flask as positive selections. The selected peptides did not bind to any of four benign cell lines that were used for binding studies. They bound to some, but not all, tumor cell lines studied and internalized into some. Another study with breast cancer cells selected for peptides that bound to HER2 receptor-positive SKBR-3 cancer cells but not HER2-negative MCF-10A benign cells [92]. The selected peptides showed homology to other ligands for HER2; however the actual cell surface target molecule was not determined. Other studies targeting a follicular thyroid carcinoma cell line [93] and colorectal tumor cell lines [73, 94] have been performed. Separate studies using the metastatic prostate cancer cell lines LNCaP (androgen sensitive) and PC-3 (androgen insensitive) selected peptides that showed no real homology between the reported sequence sets [95, 96]. As these peptide sequence families did not converge, it can be concluded that they were not binding to the same cell surface molecule. Therefore it is likely these could be used together to differentiate the two forms of prostate cancer and potentially define cell surface molecules present in one but not the other.

Outside of the use of phage display peptide libraries discussed above, recently there have been variations of the peptide MRE selection process implemented. One example is a bacterial peptide display library that has been used to select molecules that bound to breast adenocarcinoma cells ZR-75-1 but not benign cells [85]. Another variation on phage-displayed peptide panning has been performed in a microfluidic system for semiautomated selection of MREs [97]. The above examples of using peptide libraries for selecting MREs express their potential in quick and efficient screening of random molecules to obtain diagnostically or therapeutically useful tools.

### 3.2. Antibody Fragment MREs

Antibody fragment MREs have also been selected that specifically bind to tumor cells. Antibody fragment libraries are typically in the form of scFv or Fab. These libraries typically have diversity on the order of 10^9^–10^10^ molecules, thus providing great diversity from which to select monoclonal antibody-like MREs. It is likely that strong and specifically binding antibody fragments will be selected for cancer cells using differential cell SELEX.

Multiple studies have selected MREs that bind to tumor cells but not normal cells or other, nontarget tumor cells. One study has identified a scFv with very high affinity and specificity for the androgen sensitive prostate cancer cell line LNCaP but not benign or androgen insensitive prostate cell lines [75]. Two separate studies selected scFv MREs that bind to androgen insensitive prostate cancer cell lines. One targeted PC-3 cells and did negative selections with normal prostate cell lines [98]. The other targeted the C4-2B cell line, which is derived from LNCaP cells which were used as a negative target [99]. The selected antibody fragments were used for separate purposes, but the differences in selection design make it unlikely that the MREs bind to the same surface molecule. Other studies have selected scFv MREs specific for melanoma cells [100], hepatocarcinoma [101], and* ex vivo* breast cancer tissue [102]. Additionally, Fab MREs have been selected that bind to T-cell malignancies [103] and ovarian carcinoma cells [104], among others.

Antibody fragment MREs offer the specificity and pharmacological characteristics of monoclonal antibodies. Additionally, they offer the selectivity and relative ease in production provided by the differential cell SELEX process. Their multiple potential uses therefore make their selection a strong method for cancer cell studies using SELEX.

## 4. Discussion and Potential Applications

### 4.1. Surface Molecule Elucidation and Discovery

Whether the selected MRE is nucleic or amino acid based, it can be used to learn more information about its target cell. An important attribute of cell SELEX is that no* a priori* knowledge of the cell surface molecular target is necessary. It is also likely that cells in disease states will express surface molecules differently than normal cells. No previous knowledge of this differential expression is necessary however, because the differential cell SELEX process will take advantage of this fact in selecting MREs.

The cell SELEX process has been used to select MREs which aided in identification of their own surface ligand. DNA MREs have been selected that bound to tenascin C overexpressing cells and not unaltered cells [105]. As predicted, the MREs bound to tenascin C. Another study determined that ICAM-1, an intercellular adhesion molecule, was the target of scFv MREs selected on androgen insensitive prostate cancer cells [99]. DNA MREs have also been used to discover new proteins expressed on the surface of dendritic cells [106, 107]. These works show that overexpression of a surface protein does allow MRE selection against that protein. Additionally, novel or confirmatory roles of previously described molecules can be determined. There also lies the potential for discovery of new cell surface molecules or biomarkers. This use of MREs is potentially very important for a fundamental understanding of disease proteomics.

### 4.2. Disease Detection

Amino acid MREs for detection of disease are a potential clinical application of these molecules. For biomarker-based cancer detection, blood is typically extracted and subjected to immunoassays with monoclonal antibodies. There also lies the potential for magnetic, radio-, or fluorescent labeling of these antibodies for diagnostics. Antibodies used in these assays do not always bind to molecules that represent disease states. For example, screens for prostate-specific antigen (PSA) are not predictive of the presence of prostate cancer over 75% of the time [108]. This is not due to antibody specificity, but due to the poor specificity of PSA as a biomarker. Thus, these diagnostic applications make it necessary to identify MREs that bind directly to malignant cells and not to poor biomarkers that may not accurately detect the presence of disease.

Initial research using peptide MREs obtained by SELEX has been promising.* Ex vivo* human and* in vivo* mouse studies using colon cancer-specific peptides have been performed [109]. Using radiolabeled and fluorescent peptides, favorable pharmacological profiles and detection were observed. Similar results were found using an anti-prostate cancer peptide selected by phage display [110]. While not extensively explored for clinical use, peptide MREs may have great diagnostic potential.

Similar studies to those performed with peptide MREs have been done with antibody fragments [111–113]. Another potential use of antibody MREs is in* ex vivo* diagnostics such as immunoassays or microfluidic cell capture. Though using a monoclonal antibody, previous studies have shown the ability to capture circulating tumor cells with antibodies in a microfluidic device [111]. There is the obvious potential for application of antibody fragment MREs to a similar device. These and other uses make the diagnostic potential of amino acid MREs extremely important.

### 4.3. Therapeutics

Amino acids in the form of monoclonal antibodies have been widely used as therapeutics [114]. Well-known examples include trastuzumab (Herceptin: Genentech Inc.; San Francisco, CA) for HER2-positive breast cancer treatment and bevacizumab (Avastin: Genentech Inc.) that binds to vascular endothelial growth factor. These, among others, show the potential of amino acid-based therapeutic intervention.

Peptide therapeutics have been developed and used for a variety of diseases [115]. Examples of these are in infection inhibition [116], neurodegenerative disorders [117], inflammatory diseases [118], and cancer [119]. Examples of commercial peptide therapeutics include Humulin (Lilly; Indianapolis, IN) for diabetes treatment and leuprolide (Lupron: Abbott Laboratories; Abbott Park, IL) for metastatic prostate cancer treatment. Peptides obtained by cell SELEX are therefore possible alternatives to antibodies for cancer treatment.

Antibody fragments have also been used therapeutically. The humanized h5G1.1-scFv specific for complement protein C5 (pexelizumab: Alexion Pharmaceuticals; Cheshire, CT) has undergone clinical trials for its ability to reduce myocardial infarction after coronary artery bypass surgery [120]. This molecule was not selected by SELEX but was engineered from a previously obtained monoclonal antibody [121]. The results of clinical trials are mixed [122]; however the pharmacological profile of the antibody fragments is safe [123]. While these antibody fragments were engineered from monoclonal antibodies, it follows that scFv treatments obtained from SELEX will be useful and more specific for cancer treatment.

Other antibody fragments have also undergone clinical trials. A scFv molecule that binds to the HER2 receptor on the surface of tumor cells is an example. The antibody fragment was produced similar to the previous one; however it was conjugated to exotoxin A (scFv-ETA) [124]. The results of the trials proved safe, and the drug was moderately effective [125]. Additionally, an anti-digoxin Fab has been used in clinical trials for treatment of preeclampsia [126]. These uses of antibody fragments in clinically translational applications show the potential for specific drug delivery of various therapeutics or using antibody fragments as therapeutics alone.

The use of amino acid MREs is clearly a potentially fruitful field. The experimental tunability for selectivity and* in vivo* stability has been previously demonstrated. Recently, theranostics, or the dual use of single constructs for therapeutic and diagnostic purposes, has become popular [111, 127]. Those prior works demonstrate the possibility of using amino acid MREs that have been conjugated to contrast materials or nanoparticles as previously described. The use of MREs obtained by cell SELEX in therapeutics and/or diagnostics has only begun to be explored but represents great potential.

## 5. Conclusions

Selection of MREs using differential cell SELEX represents a powerful method of differentiating between cell types. This process requires no prior knowledge of cell surface molecule expression and takes advantage of differential expression profiles. Peptide and antibody fragment libraries, the host on which the library is displayed, and the selection method each have advantages and situations in which they are useful. As more MREs are being developed, it is certain that their uses and clinical investigation will continue to expand.

## Figures and Tables

**Table 1:** Types of amino acid libraries.

Library type	Advantages	Cell SELEX examples
Peptide	(i) Well-studied (ii) Widely available libraries	(i) B-cell lymphoma [89] (ii) Chronic lymphocytic leukemia [83] (iii) Non-small cell lung cancer [90] (iv) Breast cancer cell lines BT-474 & SKBR-3 [91, 92] (v) Follicular thyroid carcinoma [93] (vi) Colorectal tumor cell lines [73, 94] (vii) Metastatic prostate cancer cells [95, 96]

Antibody fragment	(i) Antibody structure and diversity selectable *in vitro* (ii) Final product can be made into a full antibody (iii) Pharmacological profile similar to antibodies which are clinically available	(i) Prostate cancer cell lines [98, 99] (ii) Melanoma [100] (iii) Hepatocellular carcinoma [101] (iv) Breast cancer tissue [102] (v) Tumor T cells [103] (vi) Ovarian carcinoma [104]

In general, advantages of both types of library include *in vivo *stability and the diversity and structure of 20 amino acids.

**Table 2:** The most common types of library display hosts.

Library host	Advantages	Examples of library construction
Phage	(i) Well-described (ii) Peptide libraries widely available	(i) Peptide libraries on M13 and T7 phage [23, 31] (ii) scFv library on M13 [33] (iii) Fab library on phage lambda [34, 35] (iv) Yeast vaccinia virus antibody fragment library [36]

Bacteria	(i) Rapid screening (ii) Use of cell sorting	(i) Peptide library on *E. coli* [38, 39] (ii) Antibody fragment on *E. coli* [40] (iii) Antibody fragment on *Staphylococcus carnosus* [41]

Yeast	(i) Posttranslational modification (ii) Use of cell sorting (iii) Fragment libraries widely available	(i) Peptide library on *Saccharomyces cerevisiae* [46] (ii) scFv library on *S. cerevisiae* [26] (iii) Fab library on *Pichia pastoris* [48]

Mammalian cells	(i) Wider array of posttranslational modifications (ii) Use of cell sorting	(i) Peptide library on T cells [56] (ii) scFv libraries on HEK-293T, T and B cells [57–59]

Ribosome/mRNA	(i) No cell transformation, thus greater library diversity (ii) Easily integrates PCR mutagenesis	Peptide and antibody fragment displayed on both ribosomes and mRNA [63, 64]

**Table 3:** Library screening methods for cell SELEX.

Method	Advantages	Disadvantages
Panning	High throughput, quick	Less powerful separation, some undesired molecules remain after washes
Magnetic separation/MACS	High throughput	Limited space if using large cells (clogging), higher failure chance compared to panning with no advantages
FACS	Very powerful separation ability with efficient tunability	Lower throughput, slower processing, expensive equipment
